# Arm Ergometry to Improve Mobility in Progressive Multiple Sclerosis (AMBOS)—Results of a Pilot Randomized Controlled Trial

**DOI:** 10.3389/fneur.2021.644533

**Published:** 2021-07-19

**Authors:** Inga Heinrich, Friederike Rosenthal, Stefan Patra, Karl-Heinz Schulz, Götz H. Welsch, Eik Vettorazzi, Sina C. Rosenkranz, Jan Patrick Stellmann, Caren Ramien, Jana Pöttgen, Stefan M. Gold, Christoph Heesen

**Affiliations:** ^1^Institute of Neuroimmunology and Multiple Sclerosis (INIMS), University Medical Center Hamburg-Eppendorf, Hamburg, Germany; ^2^Universitäres Kompetenzzentrum für Sport- und Bewegungsmedizin (Athleticum) und Institut und Poliklinik für Medizinische Psychologie, University Medical Center Hamburg-Eppendorf, Hamburg, Germany; ^3^Department of Biometry, University Medical Center Hamburg-Eppendorf, Hamburg, Germany; ^4^Department of Neurology, University Medical Center Hamburg-Eppendorf, Hamburg, Germany; ^5^APHM, Hospital de la Timone, CEMEREM, Marseille, France; ^6^Aix Marseille Univ, CNRS, CRMBM, UMR 7339, Marseille, France; ^7^Department of Psychiatry and Psychotherapy, Charité – Universitätsmedizin Berlin, Campus Benjamin Franklin, Berlin, Germany; ^8^Division of Psychosomatic Medicine, Charité – Universitätsmedizin Berlin, Campus Benjamin Franklin, Medical Department, Berlin, Germany

**Keywords:** aerobic exercise, multiple sclerosis, progressive multiple sclerosis, arm ergometry, cognition

## Abstract

**Background:** Walking disability is one of the most frequent and burdening symptoms of progressive multiple sclerosis (MS). Most of the exercise intervention studies that showed an improvement in mobility performance were conducted in low to moderately disabled relapsing–remitting MS patients with interventions using the legs. However, MS patients with substantial walking disability hardly can perform these tasks. Earlier work has indicated that aerobic arm training might also improve walking performance and could therefore be a therapeutic option in already moderately disabled progressive MS patients.

**Methods:** Patients with progressive MS and EDSS 4–6.5 were randomized using a computer-generated algorithm list to either a waitlist control group (CG) or an intervention group (IG). The IG performed a 12-week home-based, individualized arm ergometry exercise training program. Maximum walking distance as measured by the 6-min walking test (6MWT) was the primary endpoint. Secondary endpoints included aerobic fitness, other mobility tests, cognitive functioning, as well as fatigue and depression.

**Results:** Of *n* = 86 screened patients, 53 with moderate disability (mean EDSS 5.5, SD 0.9) were included and data of 39 patients were analyzed. Patients in the IG showed strong adherence to the program with a mean of 67 (SD 26.4) training sessions. Maximum work load (*P*_max_) increased in the training group while other fitness indicators did not. Walking distance in the 6MWT improved in both training and waitlist group but not significantly more in trained patients. Similarly, other mobility measures showed no differential group effect. Cognitive functioning remained unchanged. No serious events attributable to the intervention occurred.

**Conclusion:** Although maximum work load improved, 3 months of high-frequency arm ergometry training of low to moderate intensity could not show improved walking ability or cognitive functioning in progressive MS compared to a waitlist CG.

The study was registered at www.clinicaltrials.gov (NCT03147105) and funded by the local MS self-help organization.

## Introduction

Multiple sclerosis (MS) is an autoimmune inflammatory and degenerative disease of the central nervous system leading to substantial irreversible disability in 2/3 of patients (1). From a patient perspective, walking is among the three most relevant bodily functions (2). Thus, any treatment or approach to improve walking ability is highly relevant for people with MS (pwMS). Current immunotherapies have not conclusively been shown to improve impairments and benefits in progressive MS are very limited. While drug treatments such as fampridine have shown only limited effects as well (3), physiotherapy through gait and balance training is the key strategy to maintain safe mobility as long as possible (4). Rehabilitation studies have shown short-term effects on several MS symptoms, but implementing them in patients' everyday life has remained challenging (5).

In recent years, an increasing number of exercise intervention studies, using endurance exercise but also strengthening and balance trainings, has shown beneficial effects in pwMS as summarized in the systematic review by Latimer-Cheung et al. (5). However, here only 5 of 54 exercise intervention studies have explicitly addressed progressive MS. High-intensity training during short-term inpatient interventions has demonstrated impressive effects not only on mobility outcomes but also on cognitive functions in pwMS with advanced disability, i.e., EDSS 4–6 (6). In addition, a meta-analysis on exercise training in advanced MS showed significant improvement in physical fitness in pwMS with severe mobility disability in two out of five studies Edward and Pilutti (7).

In addition, MRI analysis indicated the potential of exercise interventions to slow down brain atrophy and possibly increase cortical thickness (8). Exercise intervention studies in healthy aging and early dementia indicate similar beneficial effects of exercise, although there is ongoing debate about the clinical relevance of observed effects (9). Despite encouraging in individual, small-scale studies (10), a recent meta-analysis did not find evidence that exercise improves cognitive function in MS (11).

PwMS in transition to secondary progressive MS or within the progressive phase are largely not eligible for disease-modifying drug interventions and may even be urged to stop an ineffective drug. They experience increased difficulties in mobility and thus problems to reach treatment facilities associated with fatigue, which may limit training capacity. On the other hand, continuous training three to five times/week might be the best available strategy to delay further motor impairments (5). As endurance training has gathered the most convincing evidence on beneficial effects for motor function in MS, we asked which training form might be best suitable for pwMS with walking restrictions between 100 and 500 m. In a pilot study, we compared rowing and arm ergometry with bicycle ergometry (10). While bicycle ergometry showed the strongest effects, even arm ergometry appeared to improve walking performance in this study. However, the training two to three times/week was performed at the University Medical Center Hamburg Eppendorf (UMC) only for 8 weeks. Patients perceived traveling as a substantial burden and overall training session number was moderate. Arm ergometry has rarely been studied in MS. However, in patients with substantial paraparesis, arm ergometry could offer a chance to improve fitness and muscular function. Here, we present results from a pilot study set up to clarify if home-based intensive arm ergometry study is feasible in advanced pwMS and if this approach can influence mobility, fitness, and cognition.

## Materials and Methods

### Study Design

**Figure 1:**
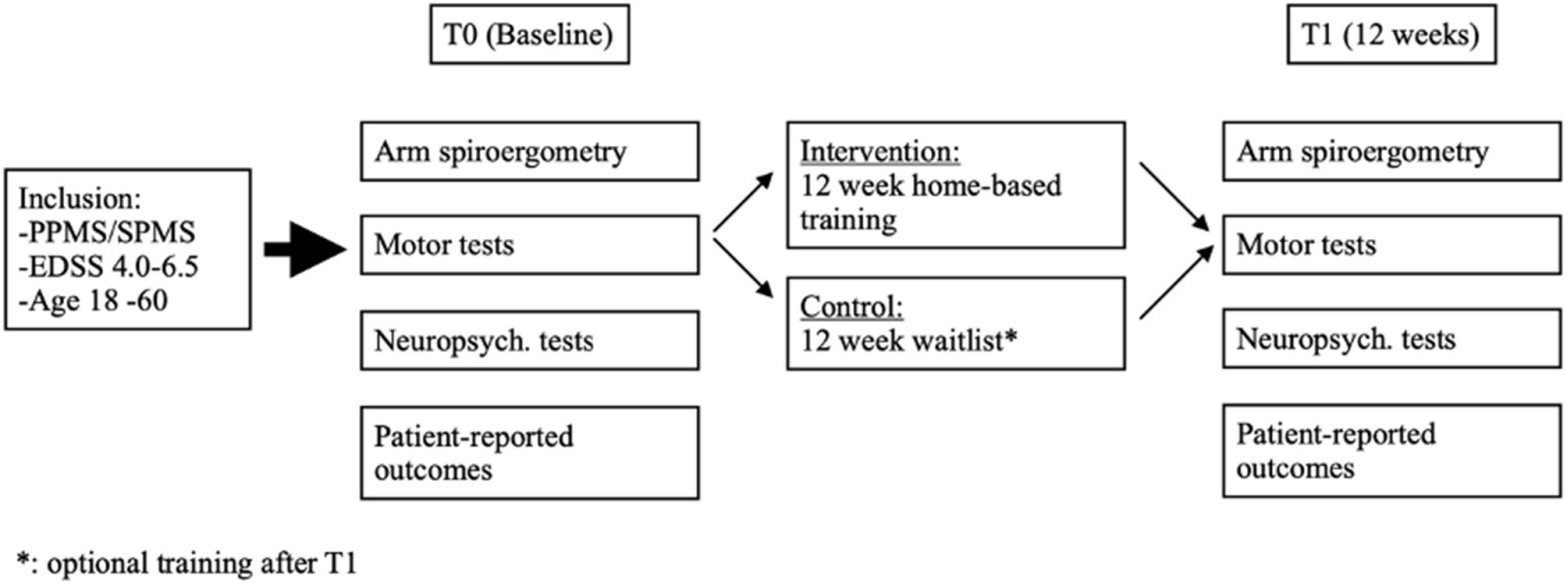
Study design: The figure shows the inclusion criteria, the examinations before randomization (T0, Baseline) to either an intervention group (IG) or a control group (CG) and the examination of both groups after 12 weeks (T1). Between T0 and T1, the IG completed a 12-week home-based training. After study completion (T1), patients of the CG were offered the same 12-week home-based training (waitlist).

The study was designed as a randomized single blinded controlled trial of a 12-week daily home-based arm ergometry exercise training program compared to a waitlist control group (CG) (see Figure 1).

### Participant Inclusion and Exclusion Criteria

Patients between the age of 18 and 60 were included if they met diagnostic criteria for clinically definite MS (12) with primary-progressive or secondary-progressive disease course and had moderate disability (Expanded Disability Status Scale EDSS 4-6.5) (13). Patients were included when they felt that they were able to perform an arm ergometry exercise training. Any medical conditions at risk during exercise training such as cardiac diseases as well a substantial cognitive deficit by clinical impression making patients presumably unable to understand and follow study rules were set as exclusion criteria. Patients with electronic implants, epilepsy, and pregnancy were excluded.

### Recruitment

Patient recruitment was conducted by screening the registry database of the MS Day Hospital at the UMC for patients who met inclusion criteria and had indicated interest in information about new clinical studies. We also recruited patients through advertisements like the website of the local self-help society German Multiple Sclerosis Society (DMSG) and leaflets in neurological practices in Hamburg.

### Randomization

Patients were consecutively randomized 1:1 using a computer-generated algorithm list. To ensure concealed allocation, the list was kept at an independent unit in another building and randomization codes were provided over the phone after determination of eligibility and assessment of baseline measures.

### Outcome Measures

The primary endpoint of the study was the 6-min walking test (6MWT) (14). Patients could use a walking aid but were instructed to use the same aid through all assessments.

Exploratory endpoints included additional mobility parameters [5-time sit-to-stand test (5SST) (15), timed tandem walk (TTW) (16), 25-foot walking test (25FWT) (17), and timed up and go test (TUG) (18)]. Furthermore, the MS walking scale 12 (MSWS-12) was used as a patient-reported measure of walking ability (19).

In addition, we included Peak oxygen consumption (VO_2peak_ in ml/min and rel. VO_2_/Bodyweight in ml/min/kg) as secondary outcome, and exploratory maximum power was also determined (*P*_max_ in W and rel. P/bodyweight in W/kg). Spiroergometry was performed with the arm crank ergometer Ergoselect 200 (Ergoline, Bitz, Germany). The ventilatory measurements were implemented with the spirometry system Metalyser-3B (Cortex Biophysik, Leipzig, Germany). Spiroergometry was performed as a ramp test with a start power of 0 W and an increase of 5 W/min. Lactate, blood pressure, and work load perception [rate of perceived exertion (RPE), Borg Scale] were measured every 2 min (20). The Borg Scale includes a score from 6 to 20 points for the subjective assessment of the effort. Blood was taken from the hyperemized earlobe with a capillary tube to measure lactate. The heart rate was measured with a heart rate monitor.

The Verbal Learning and Memory Test [VLMT; (21)] was applied as a secondary neuropsychological outcome. Exploratory neurocognitive measures were the “battery for Attentional Performance” (TAP; (22)) and other measures of the German adaptation of the BICAMS (23) such as the Brief Visuospatial Memory Test-Revised (BVMT-R) (24) and the Symbol Digit Modalities Test (SDMT) (25). Neuropsychological tests were obtained by a trained medical student (who was not blinded to group assignment).

Additional exploratory measures were obtained: Quality of life was assessed by the Hamburg Quality of Life in MS Questionnaire 10.0 (26). To measure the possible intervention impact on fatigue and depression, we used the Fatigue Scale for Motor and Cognition (FSMC) (27), and the Beck Depression Inventory (BDI-II) (28–30). The Frenchay Activity Index (FAI) (31) was obtained to measure activities of daily living. EDSS was obtained as another control measure.

Other outcomes obtained but not reported here were accelerometry data, brain atrophy, and biomarkers.

All endpoints were obtained at the Multiple Sclerosis Day Hospital at the UMC at baseline (week 0 = T0) and after the intervention (week 12 = T1) by IH, who was not blinded to the trial group of a given patient. Adherence data were recorded by the Motomed tool and/or by written documentation of patients.

### Intervention Group (IG): Individually Tailored Arm Ergometry Training

**Figure 2:**
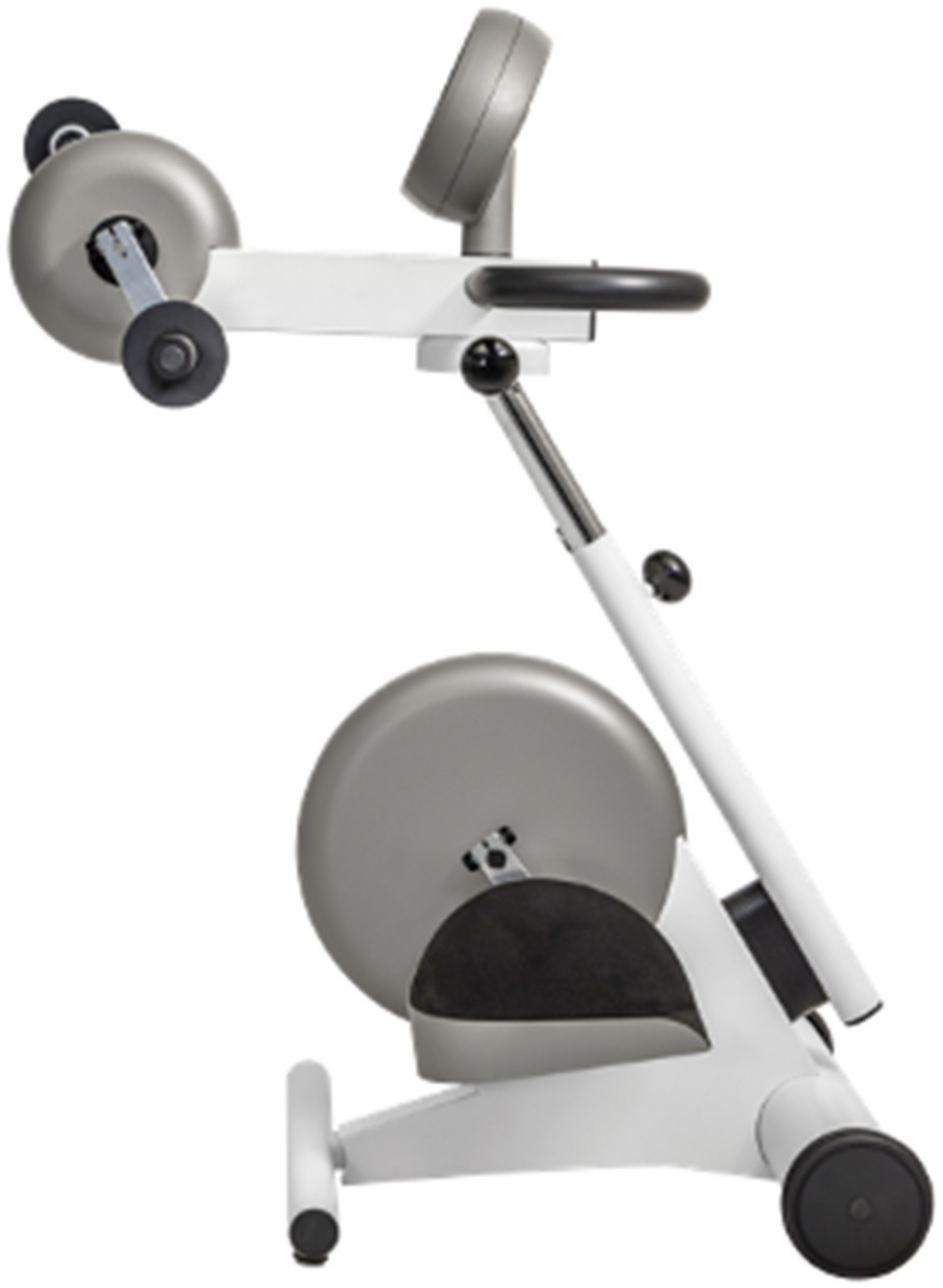
Arm ergometer (Motomed, Reck). The figure shows the arm ergometer used in the trial.

Spiroergometry results at baseline provided the anchor for the individualized training schedule. Specifically, the aerobic threshold as described earlier was determined (32). To validate the resulting training parameters, participants performed a briefing and familiarization session on the training tool Motomed (Reck, Betzenweiler, Germany) a few days later (see Figure 2). This training introduction was carried out in the Department of Sports Medicine under the supervision of trained staff. Based on the result of this session, the individual starting point for the home training plan was customized to reach the aim performing the training near the aerobic threshold level to improve aerobic capacity (33). The training concept, developed by an exercise physiologist, included one defined training interval of 6 min cranking including change of direction every minute and 2 min passive cranking by support of the device. The training plans incorporate an increase in the number of the described intervals of 8 min (see Table 1). Training at home took place for a total of 12 weeks. The overall goal was to increase both the performance (in watts) and the number of training intervals over 12 weeks.

**Table 1:** Overview of training intervals.

**Chip card**	**Week**	**Training intervals per session**			
Chip card 1	1–4	1, 2, 3, 1, 2, 3, 1	3, 1, 3, 2, 4, 2, 4	2, 5, 3, 5, 3, 5, 3	5, 3, 5, 4, 5, 4, 5
Chip card 2 (P+ 20%)	5–8	2, 4, 2, 4, 2, 5, 2	6, 3, 6, 3, 6, 3, 6	4, 6, 4, 6, 4, 6, 4	6, 5, 6, 5, 6, 5, 6
Chip card 3 (P+30%)	9–12	3, 5, 3, 5, 3, 6, 3	6, 4, 6, 4, 6, 4, 6	5, 6, 5, 6, 5, 6, 5	7, 5, 7, 5, 7, 5, 7

*One training interval on the arm ergometer consisted of 6 min load and 2 min rest. Patients received 3 chip cards for the arm ergometer with increasing power. Chip cards were exchanged every 4 weeks. Numbers listed in each column display the interval sessions for a 7-day period, where each number represents the numbers of training intervals on a given day. For example: The first day of week 2 shows indicates “3” which means that the patient has to repeat a training interval three times that day with chip card 1*.

Using the Borg Scale, the patients were able to subjectively evaluate the perceived training load. Every 4 weeks, the target performance was increased by 20%, starting from the initial performance. Subjects who rated the training to be “demanding” according to the Borg Scale (≥15 points) continued with lower performance goals and increased performance only after 4–6 or 8–10 weeks. Heart rate was continuously monitored during the training with an ear clip.

In order to increase feasibility of the training program, splitting a training session consisting of several intervals was allowed; i.e., patients were allowed to complete multiple sessions per day. The patients were asked to rate their effort on the Borg Scale for every session. The strain should be “light” to “moderate” (11–14 points) to keep the training adherence as high as possible. For the exercise training, patients used the Motomed with an integrated chipcard box for program and performance documentation. To support and supervise the patients during their home-based training, telephone calls and, if necessary, house visits were provided according to individual needs.

### Control Group (CG): Waitlist

Patients randomized to CG were offered the training intervention after the last assessment (3 months post randomization).

### Sample Size Calculation

A previous pilot study (10) had shown an increase of 63 m (SD 60.5) in the 6MWT in the arm ergometry training group compared to a decrease of 6.5 m (SD 36.0) in the non-exercising CG. We estimated at least 66% of this effect with a similar SD. To detect an expected difference of 49 m with a SD of 46 m with a power of 80% by applying 5% alpha error, 19 participants would be required in each group (NCSS-PASS, 2008). Expecting a dropout rate of up to 33%, we aimed to recruit 60 patients.

### Long-Term Follow-Up (FU) of the Cohort

All patients were telephone interviewed about a possible long-term impact of the study for their exercise behavior with self-developed Likert scale items and multiple choice items. Activity was estimated with 1 = not active, 2 = irregularly active, and 3 = regularly active. Motivation for being active (as a result of participating in the study) was rated on a scale ranging from 1 = very little to 4 = very much.

### Statistical Analysis

The primary analysis compared changes from baseline to week 12 between the IG and the waitlist CG. According to guidelines for statistical analysis of clinical trials published by The European Agency for the Evaluation of Medicinal Products (CPMP/ICH/363/96 and CPMP/EWP/2863/99), we computed the primary statistical analysis for all outcomes using ANCOVA models adjusting for baseline measurements of the respective outcome variable to evaluate treatment effects (measured as change from baseline). No other covariates were included in this primary analysis. As recommended, this model did not include treatment by covariate interactions as well. All primary analyses were conducted as intention-to-treat (ITT) including all patients who had received group allocation. Every effort was made to obtain week 12 and week 24 data from all participants (even if they dropped out of the exercise program).

In case of missing data, primary ITT analyses were conducted using a Last-Observation-Carried-Forward (LOCF) approach. In case of a significant effect in the ITT analysis, the same ANCOVA models were computed for the complete case sample as a sensitivity analysis.

### Standard Protocol Approvals, Recruitment, and Patient Consent

The trial was approved by the ethics committee of the Chamber of Physicians, City of Hamburg (Registration Number PV5408). The study was registered at www.clinicaltrials.gov (NCT03147105). Informed consent was obtained in all patients before trial inclusion.

## Results

**Figure 3:**
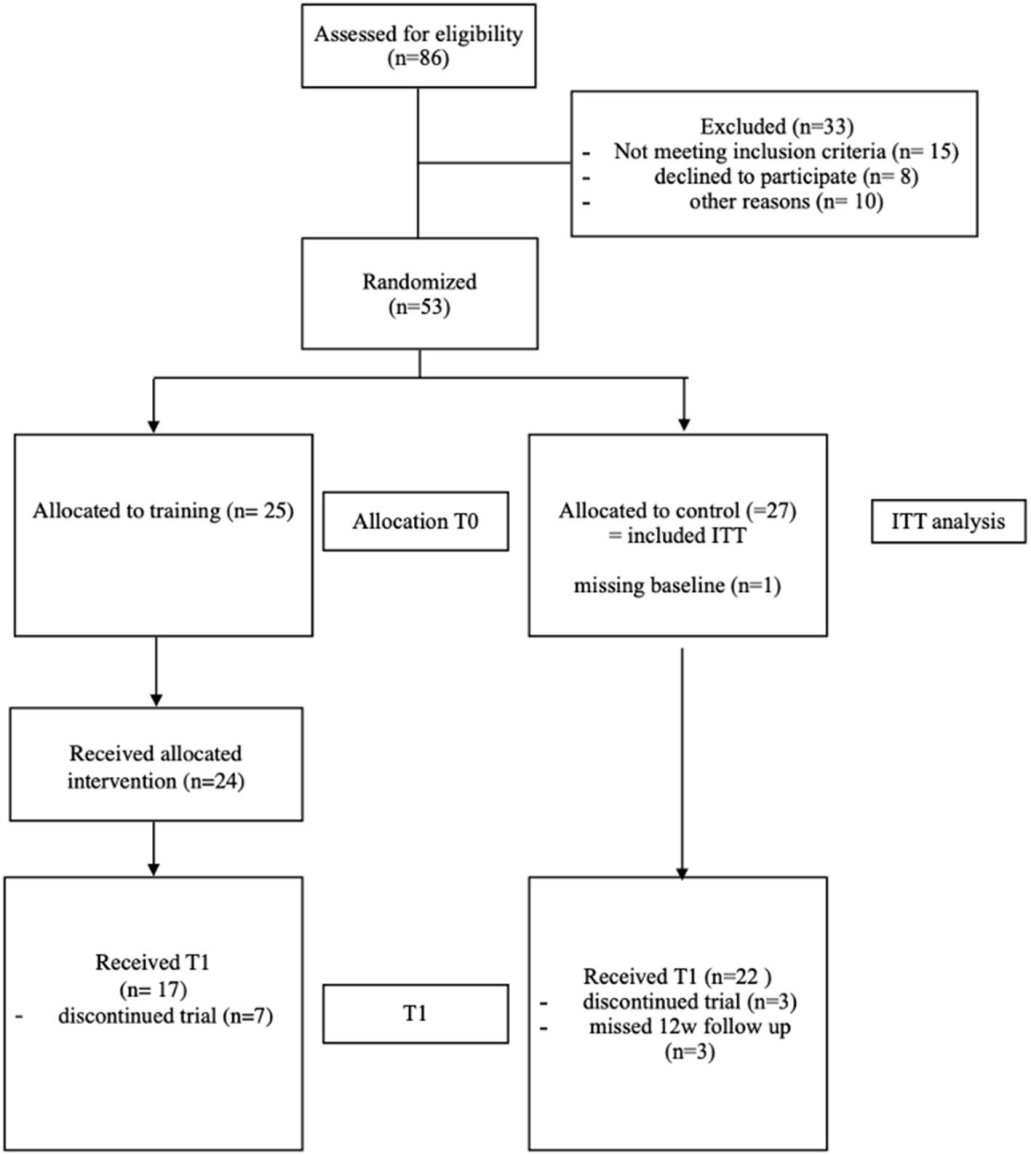
Flow chart. The figure shows the participant flow chart starting with the assessment for eligibility for participation and through to study completion.

Recruitment started in December 2017 and the trial was completed in January 2019. Eighty-six patients were interested to participate in our study. After screening for eligibility, 53 patients fulfilled the inclusion criteria (for patient attrition, see Figure 3). They were randomized 1:1 to the IG or CG. Patients in the IG completed a mean of 67 (SD 26.4) training sessions.

Forty-nine patients had complete data at baseline as well as the FU testing 12 weeks later. For the ITT analyses, all patients randomized with baseline parameters of at least the primary outcome measure were included (i.e., 25 patients in the IG and 26 in the CG). As intended, patients training intensity ratings based on the Borg Scale were around 13 (SD 1.9) on a scale from 6 to 20 points (20). Seven patients in the IG and three of the CG discontinued the study (see *Safety* section for details).

Overall, patients had moderate clinical disability (mean EDSS 5.5, SD 0.9, range 4.0–6.5) and a mean disease duration of 13.6 years (SD 6.2). Sex ratio was biased toward women (1.6:1) and both groups showed comparable distribution of demographic parameters (see Table 2). Cognitive deficits in at least one of the neuropsychological tests (defined by >1 SD below the normative data) were found in *n* = 41 participants, *n* = 20 of them in the IG. Seventeen participants (nine of them in the IG) showed three or more deficits in cognition.

**Table 2:** Clinical baseline characteristics.

	**IG**	**CG**
*N*	25	27
Age (years)	51.9 (7.9)	50.3 (6.9)
Sex (m/f)	8/17	12/15
Body weight (BMI)	22.4 (5.6)	22.9 (7.4)
Education (years)	11.7 (1.5)	11.0 (1.7)
EDSS	5.5 (0.9)	5.3 (0.9)
Disease duration (years)^*^	13.9 (6.0)	12.5 (5.2)
Disease courses (PP/SP)	5/20	5/22
On immunotherapy (%)^**^	7 (28%)	14 (52%)

*The following data were collected during the baseline assessment (week 0). Afterwards, patients were randomized using a computer-generated algorithm list to either a waitlist control group (CG) or the intervention group (IG)*.

*Data shown as mean (standard deviations)*.

* *From diagnosis*.

** *Glatirameracetat, Fingolimod, Rituximab, Alemtuzumab, Natalizumab, Teriflunomid*.

### Adherence and Feasibility

Overall training duration ranged from 10 to 48 h as documented by the Motomed and 8–48 h based on self-documentation (see Supplementary Table 1). Self-documented and Motomed documented training duration did substantially differ in some patients possibly due to difficulties in handling the chipcard reader system. Heart rate over all training sessions ranged from 87 to 109 (mean 94) while maximum heart rate ranged from 89 to 154 (mean 114). Mean Borg Scale over all training sessions ranged from 10.7 to 16.3 (mean 13.2). Six patients split training sessions throughout the day (mean 2.02 sessions/day).

### Effects on Mobility and Arm Function

**Figure 4:**
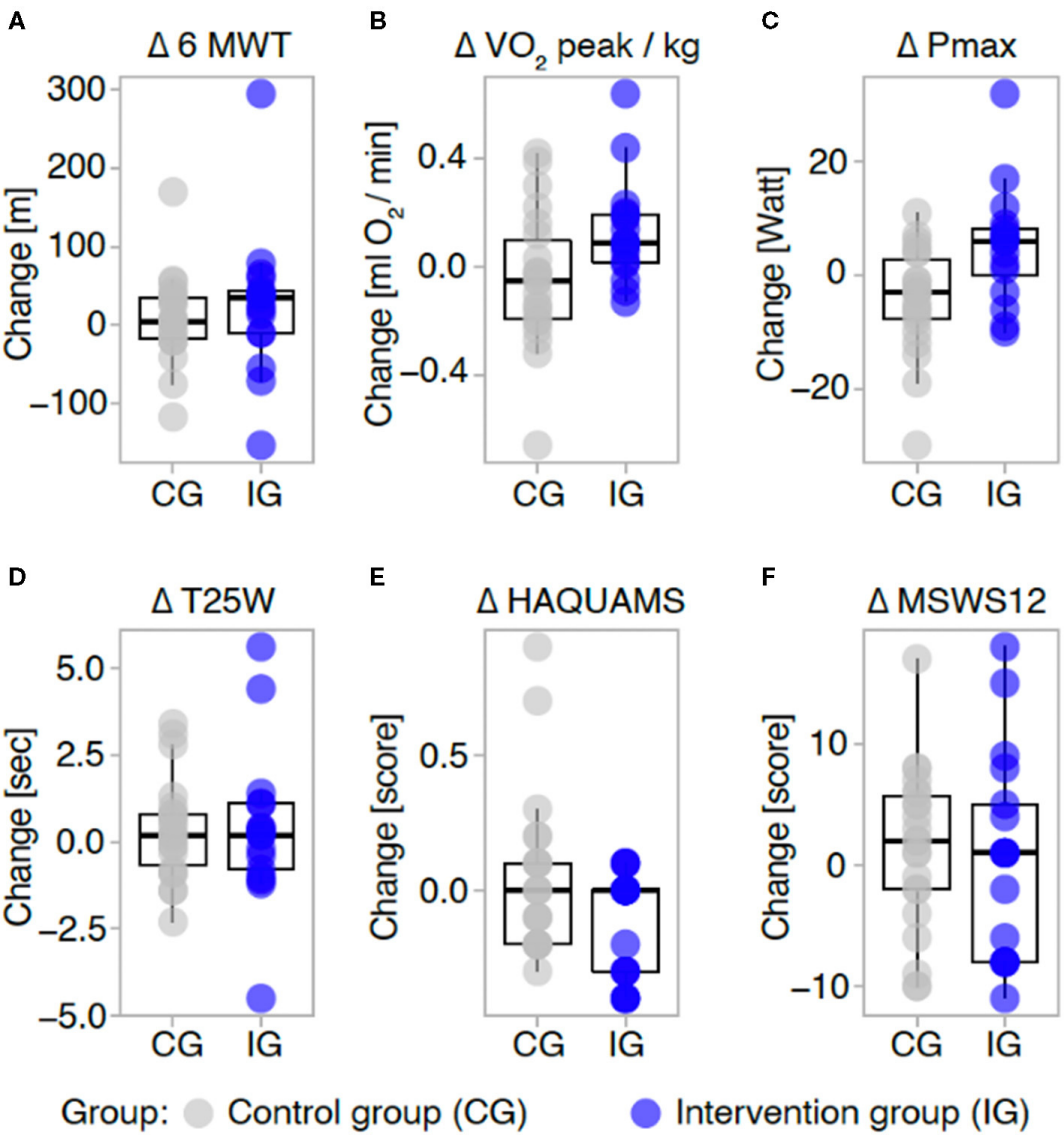
Treatment effects (complete case analyses). Difference in the main outcomes are shown as change from baseline to T1 in the control group (CG, gray) and intervention group (IG, blue). Negative values indicate a decrease from baseline to T1. Change in each group is displayed for the 6-min-walking test **(A)**, in peak oxygen consumption **(B)**, maximum power performed **(C)** 25-foot-walking-test **(D)**, quality of life measured by the hamburg Quality of Life in MS Questionnaire 10.0 (here, lower scores indicate higher QoL) **(E)** and the MS-walking-scale-12 **(F)**. Each data point depicts the change of an individual participant from baseline to T1, boxplots represent median and interquartile range. 6 MWT, Six minute walking test; VO_2_peak/kg, Peak oxygen consumption; Pmax, maximal Power, T25W, Timed 25-foot walk; HAQUAMS, Hamburger Quality of Life Questionnaire in Multiple Sclerosis; MSWS-12, 12-item MS Walking Scale.

The primary endpoint, the 6MWT, increased by 18.2 m in the IG and 7.2 m in the CG. This difference was not significant according to the ITT analysis (*p* = 0.604, ANCOVA). IG and CG showed similar performance in all exploratory motor-oriented leg and arm function tests at baseline, and none of these tests showed significant treatment effects (see Table 3). Self-reported walking ability was measured by MSWS12, indicating moderate impairment at baseline and no relevant change throughout the study. We did not observe changes in EDSS over the course of the training. To see the treatment effects the difference in the 6MWT, T25W and MSWS12 are shown as change from basline to T1 in the IG and the CG (see Figure 4).

**Table 3:** Motor, fitness, and patient reported outcomes (ITT analyses with last-observation carried forward).

	**IG**		**CG**		***p***
	**Baseline**	**Week 12**	**Baseline**	**Week 12**	
**Motor function and aerobic fitness**					
6MWT (m)	231.2 (106.6)	249.4 (124.9)	275.0 (102.6)	282.9 (115.3)	0.604
T25W (sec)	12.5 (10.1)	12.7 (10.2)	10.5 (12.1)	10.7 (12.8)	0.827
5SST (sec)	22.4 (9.4)	20.5 (7.8)	19.3 (11.3)	19.4 (11.7)	0.180
TTW (sec)	15.3 (6.6)	14.8 (6.9)	15.8 (8.8)	15.7 (8.9)	0.726
TUG (sec)	15.5 (8.4)	15.8 (9.2)	14.8 (13.9)	14.2 (11.8)	0.259
9HPT right (sec)	27.9 (8.2)	29.6 (14.6)	28.2 (12.6)	28.8 (13.0)	0.618
9HPT left (sec)	28.8 (7.8)	30.1 (12.8)	31.8 (20.2)	35.7 (39.9)	0.981
VO_2_peak/kg (ml O_2_/min)	1.01 (0.26)	1.1 (0.26)	1.20 (0.36)	1.16 (0.34)	0.174
Pmax (Watt)	47.5 (13.7)	51.0 (12.9)	53.80 (15.9)	50.08 (11.8)	**0.021**
EDSS	5.5 (0.9)	5.5 (0.8)	5.3 (0.9)	5.4 (0.9)	0.344
**Patient-reported outcome measures**					
BDI	9.4 (7.4)	8.8 (6.0)	10.9 (7.5)	11.9 (8.0)	0.091
FSMC	68.8 (13.2)	68.1 (17.1)	71.7 (11.5)	72.8 (11.7)	0.410
MSWS12	34.0 (8.1)	34.5 (7.2)	32.5 (8.9)	33.7 (9.7)	0.711
**HAQUAMS**					
Lower ex.	3.5 (0.7)	3.3 (0.8)	3.3 (0.8)	3.3 (0.7)	0.41
Upper ex.	2.2 (0.6)	2.3 (0.6)	1.9 (0.5)	1.9 (0.6)	0.376
Fatigue	2.8 (1.0)	2.6 (0.9)	3.0 (0.9)	3.0 (0.9)	0.149
Thinking	2.5 (1.0)	2.4 (1.0)	2.8 (0.9)	2.8 (0.9)	0.626
Comm.	2.2 (0.7)	2.2 (0.7)	2.0 (0.9)	2.2 (0.9)	0.092
Mood	2.7 (0.7)	2.5 (0.7)	2.6 (0.9)	2.6 (0.9)	0.165
Total	2.7 (0.4)	2.6 (0.4)	2.6 (0.5)	2.6 (0.5)	**0.045**
FAI (total)	29.0 (8.5)	28.2 (9.5)	33.3 (8.5)	32.4 (9.0)	0.919

*The table shows results of motor function, aerobic fitness, and patient-reported outcome measures at baseline (week 0) and after 12 weeks both in the intervention group (IG) and the control group (CG). Mean values and standard deviation (in brackets) are shown. P-values are shown on the right according to ANCOVA. The bold values show significance*.

*6 MWT, Six minute walking test; 9 HPT, Nine-Hole Peg Test; T25FW, Timed 25-foot walk; P_max_, maximal Power; BDI, Beck Depression Inventory; FSMC, Fatigue Scale for Motor and Cognitive Functions; MSWS-12, 12-item MS Walking Scale; HAQUAMS, Hamburger Quality of Life Questionnaire in Multiple Sclerosis, higher scores indicate a worse quality of life; FAI, Frenchay Activity Inventary*.

### Effects on Fitness Parameters

Although not significant, there was a trend toward a decrease of the VO_2_peak/kg CG and toward an increase in the IG (see Figure 4). Similarly, maximum power started from a higher level in CG but declined for 3.7 W while maximum power increased for 3.5 W in the IG. This effect was small but significant (ANCOVA, *p* = 0.021).

### Effects on Quality of Life, Fatigue, Depression, and Daily Functioning

MS-specific quality of life improved in the IG compared to the CG, although the change again was small (0.1 points in IG and 0 points in CG, *p* = 0.045, ANCOVA) and could not be detected in subscales (see Table 3 and Figure 4). Correspondingly, there was an improvement in depression ratings in the BDI although this was just a trend finding (ANCOVA *p* =0.091). However, fatigue ratings that showed modest impairment at baseline were not altered throughout the study. Daily functioning as measured by the FAI in general did not change through the 3 months study period.

### Effects on Cognitive Function

Measures of verbal learning, information processing and memory, spatial learning and memory, as well as attention remained stable throughout the study period (see Table 4).

**Table 4:** Cognitive outcomes (ITT analyses with last-observation carried forward).

	**IG**		**CG**		***p***
	**Baseline**	**Week 12**	**Baseline**	**Week 12**	
SDMT (points)	44.6 (15.4)	45.7 (14.3)	43.2 (16.7)	44.6 (13.2)	0.970
VLMT 1-5 learning (points)	52.5 (8.6)	54.4 (8.7)	51.2 (12.0)	53.0 (12.5)	0.868
VLMT 5-7 delayed memory (points)	2.2 (2.1)	1.5 (1.8)	2.2 (2.4)	1.9 (2.4)	0.494
BVMT-R total learning (points)	22.5 (7.9)	24.1 (6.5)	20.0 (9.1)	21.4 (8.5)	0.908
BVMT-R recall (points)	8.5 (2.9)	8.9 (2.9)	7.8 (3.4)	8.3 (2.8)	0.821
TAP Tonic Alertness (msec)	317 (71.1)	309 (63.0)	287 (64.1)	286 (61.8)	0.400
TAP Phasic Alertness (msec)	317 (83.7)	297 (65.9)	300 (64.2)	290 (65.8)	0.326

*The table shows cognitive function at baseline (week 0) and after 12 weeks both in the intervention group (IG) and the control group (CG). Mean values of raw scores and standard deviation (in brackets) are shown. P-values are shown on the right, based on the primary analysis (ITT ANCOVA)*.

*SDMT, Symbol Digit Modalities Test; VLMT, verbal learning and memory test; BVMT-R, Brief Visuospatial Memory Test-Rrevised; TAP tonic alertness, Test battery for attention tonic alertness; TAP phasic alertness, Test battery for attention phasic alertness*.

### Safety

At T1 (at 3 months) and T2 (at 6 months), patients reported no intervention-related injuries such as falls or accidents. General worsening of the overall condition was reported by one patient in the IG, which was not mirrored in EDSS change. One patient experienced a lower leg fracture due to an accident, which was not related to the training but led to the interruption of the study. Another patient developed neck–shoulder pain during the training, which resolved in the FU. Besides that, one patient developed pyelonephritis and had to stay in the hospital for a while, and another one started in-patient MS rehabilitation. Two patients had no motivation to exercise over 12 weeks and reported that the training took too much time.

Two patients of the CG noted that the waiting period was too long and some indicated decreasing motivation in the 12 weeks.

### Long-Term FU

A telephone-administered survey 1–2 years after the original study asked patients about their activity level before the study and thereafter. Sixteen patients (94%) from 17 who participated in the IG could be contacted. Based on the applied three-point Likert scale, the IG turned from a level of being not regularly active (mean 2.1, SD 0.8) to a mean of being regularly active (2.7, SD 0.6). To maintain being active, all 16 respondents would have wished a telephone FU after the study. Nine participants indicated on request that a web-based interactive platform might be helpful. Also, all would recommend the study to other pwMS after having terminated the study.

## Discussion

Although pwMS show substantial limitations in doing leg-based endurance exercise when they have entered the progressive phase of the disease, limited research has explored the potential of aerobic arm training in these patients. Based on an earlier trial (10), we conducted a pilot study for arm ergometry in pwMS. In contrast to previous center-based interventions (10, 32), AMBOS aimed to establish a high-frequency home-based training.

This study showed that a high frequency at home training is feasible for 3 months with a substantial number of 67 sessions. Of note, this trial enrolled a cohort of progressive pwMS with a mean EDSS of 5.5 referring to being able to walk 100 m without aid. As we included patients with an EDSS up to 6.5, more advanced disability in some patients might have contributed to the overall lack of a detectable treatment effect.

However, our trial clearly missed its primary endpoint as we could not detect treatment effects on our pre-specified measures of walking ability. Was the goal of improving walking by arm training too ambitious? Upper body training is firmly established in spinal cord injury showing improved aerobic capacity and also functional benefit in enhanced wheelchair handling (34, 35). It has been argued that high training intensity cannot be achieved with upper limb training (36). This has been attributed to less muscle mass than in lower limb training. However, studies comparing hand and leg cycling ergometry in healthy female individuals have shown that after 7 weeks of training, 75% of VO_2_ peak compared to leg ergometry can be reached (36). Central adaptations as mirrored in an increase in cardiac output and oxygen delivery to the muscles can be shown after upper body endurance training (37). However, leg cycling performance was not improved in the study of Hettinga et al. (36), indicating that systemic adaption based on arm training might not be sufficient to improve aerobic training performance in the legs.

On the other hand, work in elderly people has shown that walking performance in patients with intermittent claudication (~70 years) and in patients with peripheral arterial disease improved after upper body endurance training, at least partly due to a better lower limb O_2_ delivery (38, 39). Although pathomechanisms in spinal cord injury and claudication are very different from MS, these data are in line with putative systemic effects of arm ergometry. Thus, arm training might show transfer effects in low-intensity tasks such as walking in people with a low fitness level. Future studies directly comparing arm vs. leg ergometry may be informative regarding the possibility of differential effects on walking ability.

Having said that, it should be noted that arm ergometry training programs can differ substantially. Our arm cycling spiroergometry as well as the training tool in our earlier study needed more trunk and shoulder work, possibly explaining the minor impact of the training despite the high volume of sessions.

While most secondary endpoints also showed no significant treatment effects, we did observe some promising signals with regard to increases in fitness indices in the training group, particularly in *P*_max_. This effect was small, but mirrored the findings in our earlier study (10).

As inpatient high-intensity training in pwMS with advanced disability has been shown not only to be feasible but also to show stronger effects than standard aerobic training (6), even a higher training burden might be feasible for example in a closer supervised initial phase of a longer-term training concept.

Although our patient cohort trained on their own with an elaborate chip-card training program, a substantial amount of telephone coaching was necessary to keep the patients adherent. Most of them received telephone coaching at the beginning, after 4 weeks, and after 8 weeks. For special questions, patients could call or write an email at any time. Our adherence data indicate that some patients had difficulties with the treatment documentation. Splitting of the daily program into a number of short sessions might have limited putative training effects in that subgroup.

Therefore, we believe that any approach in progressive MS stages needs a substantial amount of education and continued personal support.

Qualitative evaluations are not standard in MS exercise trials, and our data on these aspects are also limited. Future studies might thus benefit from more detailed patient feedback to gain the most information from pilot and feasibility studies on exercise interventions (40).

Although the arm ergometry approach was justified by our previous study (10), applying the intervention in a home-based setting added another variable of uncertain weight to the experimental approach.

Our study cohort was small and baseline data of 6MWT, 25FWT, and EDSS indicate a worse mobility status in the IG, which might have blurred a possible differential effect of the intervention. A longer trial duration might have led to stronger effects. The long-term FU data of our study showed that more patients became active through the trial and that they would recommend the study to other pwMS, indicating that such an extension might be feasible. Drug trials aiming to slow progression hardly manage to show this effect through at least 2 years trial duration and several hundred patients (41). Most work on exercise intervention studies in progressive MS were administered in a rehabilitation or outpatient setting but not attached to the daily lives of patients. Therefore, we believe that longer durations are needed to show if exercise can slow progression or even increase fitness and functioning. In fact, an exercise training approach in progressive MS needs to aim for a lifelong implementation, which might even need adaptation when the disease worsens despite all efforts.

In contrast to earlier work (10), we could not show improved performance on cognitive measures of verbal learning and attention. Current evidence on exercise training on cognition in MS does not indicate a benefit as summarized by a recent meta-analysis (11). However, another small short-term study in RRMS with low disability but significant cognitive impairment could show some amelioration of deficits, although improvements compared to controls could only be shown in two out of seven neurocognitive measures (42).

Finally, although most current data exist with regard to aerobic training, a combination of aerobic training with resistance training or HIT training might be more potent to affect brain functioning as measured by cognitive tests (43).

## Conclusion

Taken together, our pilot RCT indicates that home-based training in progressive MS is feasible, but the current format seems too weak in intensity and overall duration to justify any valid conclusion on the effect of especially arm aerobic exercise on mobility and cognitive performance in progressive MS.

## Data Availability Statement

The raw data supporting the conclusions of this article will be made available by the authors, without undue reservation.

## Ethics Statement

The studies involving human participants were reviewed and approved by the Ethical Committee of the Board of Physicians in the State of Hamburg. The patients/participants provided their written informed consent to participate in this study.

## Author Contributions

CH conducted, designed, and supervised the study. IH and FR conducted the examinations, collected the data and supervised the patients, wrote the manuscript with CH. SR supervised and supported the evaluation. JP supervised and analyzed the neuropsychological examinations. SP carried out the sport medicine examinations. SG and EV analyzed the data. GW, K-HS, and JS supervised the study. CR was involved in extracting additional data from the database and developing graphical presentations. All authors discussed and contributed to the manuscript.

## Conflict of Interest

The authors declare that the research was conducted in the absence of any commercial or financial relationships that could be construed as a potential conflict of interest.
