# Supplementary material for: Arm Ergometry to Improve Mobility in Progressive Multiple Sclerosis (AMBOS)—Results of a Pilot Randomized Controlled Trial
Source: Front Neurol. 2021 Jul 19;12:644533. doi: 10.3389/fneur.2021.644533 (PMC8326796; doi:10.3389/fneur.2021.644533)
Supplement: Supplementary file 1 [file Table_1.docx]

**Suppl Table 1.** Overview of adherence data

|  | **number of sessions (motomed)** | **number of sessions**  **(self-documented)** | **training duration (motomed)** | **training duration (self-documented)** | **heart rate (mean)** | **maximum heart rate (mean)** | **Borg scale (mean)** |
| --- | --- | --- | --- | --- | --- | --- | --- |
| 1 | 98 | md | 17:43:06 | 48:20:00 | 87 | 106 | 15,8 |
| 2 | 87 | md | 48:25:46 | 31:00:00 | 94 | 108 | 10,7 |
| 3 | 93 | md | 38:05:22 | 37:35:00 | 93 | 107 | 15,3 |
| 4 | 90 | md | 40:07:58 | md | 106 | 121 |  |
| 5 | 25 | 78 | 10:36:02 | 31:50:00 | 95 | 105 | 12,3 |
| 6 | 86 | md | 35:57:00 | 35:41:00 | 93 | 104 | 13,7 |
| 7 | 80 | md | 33:38:12 | 33:40:00 | 88 | 101 | 15,7 |
| 8 | 64 | md | 31:27:02 | 35:20:00 | 104 | 117 | 10 |
| 9 | 85 | md | 16:59:31 | 16:21:00 | 109 | 138 | 13 |
| 10 | 86 | md | 36:32:11 | 36:17:00 | 83 | 91 | 11,2 |
| 11 | 81 | md | 35:57:00 | 33:00:00 | 81 | 89 | md |
| 12 | 73 | md | md | 30:00:00 | 94 | 106 | 16,3 |
| 13 | md | 83 | md | 31:14:00 | 93 | 119 | 13 |
| 14 | md | 57 | md | md | md | md | 13 |
| 15 | md | 35 | md | 08:07 | 91 | 119 | 12,7 |
| 16 | md | 65 | md | 22:10:00 | 100 | 154 | 14,3 |
| 17 | md | md | md | md | md | md | md |
| dropout | md | md | md | md | md | md | md |
| dropout | md | md | md | md | md | md | md |
| dropout | md | md | md | md | md | md | md |
| dropout | md | 43 |  | 13:50:00 | 94 | 132 | 11,7 |
| dropout | md | md | md | md | md | md | md |
| dropout | md | md | md | md | md | md | md |
| dropout | 4 | md | 00:17:34 | md | 97 | 112 | md |
| dropout | md | 38 | md | 12:35:00 | 98 | 123 | md |

Overall training duration ranged from 10 hours to 48 hours as documented by the motomed and 8 to 48 hours based on self-documentation. Self-documented and motomed documented training duration did substantially differ in some patients possibly due to difficulties in handling the chipcard reader system. Heart rate over all trainings ranged from 87-109 (mean 94) while maximum heart rate ranged from 89-154 (mean 114). Mean Borg scale over all trainings ranged from 10.7-16.3 (mean 13.2). 6 patients did split training sessions through the day (median 27 sessions). md=missing data
