# Supplementary material for: Arm Ergometry to Improve Mobility in Progressive Multiple Sclerosis (AMBOS)—Results of a Pilot Randomized Controlled Trial
Source: Front Neurol. 2021 Jul 19;12:644533. doi: 10.3389/fneur.2021.644533 (PMC8326796; doi:10.3389/fneur.2021.644533)
Supplement: Supplementary file 2 [file Data_Sheet_1.PDF]

Two-Sample T-Test Power Analysis

Page/Date/Time 1 27.06.2016 14:52:47

Numeric Results for Two-Sample T-Test

Null Hypothesis: Mean1=Mean2. Alternative Hypothesis: Mean1<>Mean2
The standard deviations were assumed to be unknown and equal.

Power	N1	N2	Allocation Ratio	Alpha	Beta	Mean1	Mean2	S1	S2
0.80388	19	19	1.000	0.05000	0.19612	46.000	0.000	49.000	49.000

References

Machin, D., Campbell, M., Fayers, P., and Pinol, A. 1997. Sample Size Tables for Clinical Studies, 2nd Edition. Blackwell Science. Malden, MA.
Zar, Jerrold H. 1984. Biostatistical Analysis (Second Edition). Prentice-Hall. Englewood Cliffs, New Jersey.

Report Definitions

Power is the probability of rejecting a false null hypothesis. Power should be close to one.
N1 and N2 are the number of items sampled from each population. To conserve resources, they should be small.
Alpha is the probability of rejecting a true null hypothesis. It should be small.
Beta is the probability of accepting a false null hypothesis. It should be small.
Mean1 is the mean of populations 1 and 2 under the null hypothesis of equality.
Mean2 is the mean of population 2 under the alternative hypothesis. The mean of population 1 is unchanged.
S1 and S2 are the population standard deviations. They represent the variability in the populations.

Summary Statements

Group sample sizes of 19 and 19 achieve 80% power to detect a difference of 46.000 between the null hypothesis that both group means are 46.000 and the alternative hypothesis that the mean of group 2 is 0.000 with estimated group standard deviations of 49.000 and 49.000 and with a significance level (alpha) of 0.05000 using a two-sided two-sample t-test.

Two-Sample T-Test Power Analysis

Page/Date/Time 2 27.06.2016 14:52:47

Chart Section
